# Association of *VDR* gene polymorphisms with prediabetes and Type 2 diabetes mellitus in a sample of the Iranian population

**DOI:** 10.1371/journal.pone.0339758

**Published:** 2026-02-09

**Authors:** Dorsa Salabat, Nekoo Panahi, Noushin Fahimfar, Masoud Saeedi, Hanieh Radkhah, Abbasali Keshtkar, Hamid Reza Aghaei Meybodi, Afshin Ostovar, Bagher Larijani, Mahsa M. Amoli

**Affiliations:** 1 Osteoporosis Research Center, Endocrinology and Metabolism Clinical Sciences Institute, Tehran University of Medical Sciences, Tehran, Iran; 2 Metabolic Disorders Research Center, Endocrinology and Metabolism Molecular-Cellular Sciences Institute, Tehran University of Medical Sciences, Tehran, Iran; 3 Department of Endocrinology, Shariati Hospital, Tehran University of Medical Sciences, Tehran, Iran; 4 Department of Epidemiology and Biostatistics, School of Public Health, Tehran University of Medical Sciences, Tehran, Iran; 5 Internal Medicine Department, School of Medicine, Sina Hospital, Tehran University of Medical Sciences, Tehran, Iran; 6 Department of Disaster and Emergency Health, School of Public Health, Tehran University of Medical Sciences, Tehran, Iran; 7 Evidence based Research Center, Endocrinology and Metabolism Clinical Sciences Institute, Tehran University of Medical Sciences, Tehran, Iran; 8 Endocrinology and Metabolism Research Center, Endocrinology and Metabolism Clinical Sciences Institute, Tehran University of Medical Sciences, Tehran, Iran; Karmanos Cancer Institute, Wayne State University School of Medicine, UNITED STATES OF AMERICA

## Abstract

**Background:**

Type 2 diabetes mellitus (T2DM) is a prevalent chronic disorder responsible for most diabetes cases. The role of *vitamin D receptor* (*VDR*) gene variants in T2DM susceptibility has been investigated previously; however, the results are contradictory, particularly among diverse ethnic groups. This study aimed to investigate the association between *VDR* gene polymorphisms and T2DM in a sample of the Iranian population.

**Methods:**

Data from 976 participants of the phase 3 Iranian Multicenter Osteoporosis Study (IMOS) were analyzed. Five *VDR* polymorphisms (ApaI, TaqI, EcoRV, FokI, and BsmI) were genotyped and assessed among diabetic (DM), prediabetic (preDM), and healthy participants. We employed logistic regression models to evaluate the association of *VDR* polymorphisms with preDM and DM, accounting for potential confounding factors, including age, gender, body mass index (BMI), and vitamin D levels.

**Results:**

The multivariable logistic regression analysis indicated a significant relationship between the ApaI polymorphism (rs7975232) and the risk of developing preDM or DM. Specifically, individuals with the *CC* genotype exhibited a 66% increased likelihood of progressing to preDM or DM compared to those with the *AA* genotype [OR: 1.66 (1.07, 2.56)]. In examining the *A* dominant state, individuals carrying at least one *A* allele (*AA* or *AC* genotypes) were found to be 35% less likely to develop preDM or DM when compared to those with the *CC* genotype [OR: 0.65 (0.43, 0.97)]. No significant associations were identified for the other polymorphisms (TaqI, EcoRV, FokI, and BsmI).

**Conclusion:**

These findings indicate that the *C* allele of the ApaI polymorphism may be associated with an increased susceptibility to T2DM in this population, underscoring the *VDR* gene’s role in T2DM risk and pointing to potential personalized prevention and management strategies.

## Introduction

Type 2 diabetes mellitus is a common chronic disorder accounting for most diabetes cases with a prevalence of 14.6% in the Middle East [1]. It is mainly caused by insulin resistance and impaired insulin secretion and is associated with complications such as nephropathy, neuropathy, retinopathy, and cardiovascular diseases, can cause a substantial burden on individuals and healthcare systems globally [2,3]. An interplay between genetic, epigenetic, and environmental factors can lead to type 2 diabetes mellitus, making it a multifactorial disease [4].

Low vitamin D levels have been associated with impaired glucose tolerance and developing type 2 diabetes mellitus [5]. The mechanism is still unknown but might involve vitamin D affecting the secretion of insulin from β-cells and improving insulin resistance [5,6]. Vitamin D exerts its function by binding to the vitamin D receptor (*VDR*), which is a cytosolic/nuclear receptor belonging to the steroid/thyroid hormone receptor family [7]. The *VDR* gene is found in various cell types throughout the body and regulates the expression of numerous genes related to bone metabolism, immune system activity, and organ development [8–10].

The *VDR* gene is located on chromosome 12q13.11 and has several polymorphisms with some of the more studied single-nucleotide polymorphisms (SNPs) including FokI (rs2228570), TaqI (rs731236), ApaI (rs7975232), and BsmI (rs1544410) [11]. Several studies carried out on different populations and ethnicities have investigated the association of these polymorphisms with the incidence of type 2 diabetes mellitus and found different results, with some showing an association [12–15] while others do not [16,17]. A recent meta-analysis and following studies have shown varying associations between *VDR* gene polymorphisms and the disease susceptibility, with significant ethnic variations observed [18,19]. In Iran, only a few studies have examined VDR polymorphisms in relation to type 2 diabetes mellitus, with small sample sizes, limited polymorphisms, and conflicting findings, highlighting the complexity of these genetic associations and the necessity of larger-scale studies [20–23]. Moreover, data on the EcoRV (rs4516035) polymorphism are extremely limited, with no comprehensive study conducted in the Iranian population.

Given the heterogeneous findings in different ethnic groups and the Iranian population, in this study, we aim to evaluate the possible association between genetic susceptibility to type 2 diabetes mellitus and five *VDR* gene polymorphisms, including FokI, TaqI, ApaI, BsmI, and a less studied polymorphism, EcoRV (rs4516035), in the adult Iranian population. Our study has one of the largest populations among the studies conducted in Iran and the Middle East.

## Method

### Study population

This study included participants from phase III of the Iranian Multicenter Osteoporosis Study (IMOS-3), conducted in the urban areas of Arak and Sanandaj in winter 2011. IMOS-3 aimed to assess the prevalence of osteoporosis, vitamin D deficiency, and related risk factors across Iranian urban populations [24,25]. A total of 2,100 adults aged 20 years and older were enrolled using a randomized clustered sampling method. Exclusion criteria and detailed study procedures have been described previously [24,25]. To ensure the accuracy and relevance of the findings related to bone health, several exclusion criteria were applied. Participants who were non-Iranian, recent city residents (less than one year), or those with mental or psychological disorders, as well as individuals unable to cooperate with interviewers, were excluded. Additionally, those with deformities affecting bone mineral density (BMD) measurements, individuals weighing over 120 kg, and those with prolonged hospitalization or immobilization were not included. Further exclusions encompassed participants with infertility, severe chronic illnesses such as renal or liver failure, cancer, chronic diarrhea, malabsorption, or recent vitamin D supplementation [24,25]. Competitive ELISA using enzyme immunoassay was used for measuring the level of 25(OH) vitamin D. Participants with available data on diabetes status and genotyping were included in the present study. Those with type 1 diabetes were excluded.

### Genotyping

Genotyping of VDR gene polymorphisms was performed exclusively on blood samples from Sanandaj participants. Genomic DNA was extracted using the phenol-chloroform method. Genotyping was performed using ABI Taqman Genotyping assay specific for each SNP and Restriction fragment length polymorphism (RFLP). Genotyping accuracy was confirmed by Sanger sequencing. Further methodological details are available in the IMOS-3 protocol [25].

The genetic study of VDR gene polymorphisms was conducted using whole blood samples from Sanandaj participants, which had been previously collected and stored in EDTA tubes at −70°C following ethical approval granted on July 9, 2014. Genomic DNA was extracted using the phenol-chloroform method. Subsequently, after obtaining ethical approval for the present study on May 29, 2024, we accessed de-identified IMOS-3 data from the Endocrinology and Metabolism Research Institute for research purposes. Data cleaning was subsequently performed. The authors did not have access to any information that could identify individual participants during or after data collection. For the current analysis, only participants from Sanandaj with available information on VDR gene polymorphisms and type 2 diabetes mellitus statuses were included. Out of the 1268 Sanandaj participants, 223 had no data on *VDR* polymorphisms, and 266 lacked sufficient and consistent data on the disease status (with some overlap in missing data, meaning that some individuals lacked sufficient data on both diabetes status and *VDR* polymorphisms). Individuals with type 1 diabetes mellitus were also excluded. After applying these criteria, 976 participants with available data on type 2 diabetes mellitus status, and with assessment of at least one of the *VDR* gene polymorphisms, remained for analysis.

Ethical approval for the IMOS study was obtained from the Ethics Committees at the Endocrinology and Metabolism Research Institute of Tehran University of Medical Sciences. The ethical code of the current study is IR.TUMS.SINAHOSPITAL.REC.1403.035. Informed consent was obtained from all subjects in the original study and the study was conducted in accordance with the Declaration of Helsinki.

### Variables

Type 2 diabetes mellitus (T2DM): Participants with fasting blood sugar (FBS) ≥126 mg/dl or glycated hemoglobin (HbA1C) ≥ 6.5% or history of T2DM accompanied by using antidiabetic medication were considered diabetic (DM); those with 126 > FBS ≥ 100 mg/dl and 6.5 > HbA1C≥5.6% were deemed prediabetic (preDM). All other participants were categorized as healthy.

The genotypes for the various polymorphisms in the *VDR* gene are classified as follows: ApaI (rs7975232): *AA*, *AC*, *CC*; TaqI (rs731236): *TT*, *TC*, *CC*; FokI (rs2228570): *CC*, *CT*, *TT*; EcoRV (rs4516035): TT*, CT*, *CC*; BsmI (rs1544410): *GG*, *GA*, *AA.*

*VDR* polymorphism carrier states: For each polymorphism, carrier states were defined as follows: For ApaI, *A* carrier (*AA* + *AC*) and non-carrier (*CC*), and *C* carrier (*CC* + *AC*) and non-carrier (*AA).* For TaqI, *T* carrier (*TT* + *TC*) and non-carrier (*CC*), and *C* carrier (*CC* + *TC*) and non-carrier (*TT*). For FokI, *C* carrier (*CC* + *CT*) and non-carrier (*TT*), and *T* carrier (*TT* + *CT*) and non-carrier (*CC*). For EcoRV, *T* carrier (*TT* + *CT*) and non-carrier (*CC*), and *C* carrier (*CC* + *CT*) and non-carrier (*TT*). For BsmI, *G* carrier (*GG* + *GA)* and non-carrier (AA*),* and *A* carrier (*AA* + *GA*) and non-carrier (*GG*).

### Statistical analysis

Continuous variables with a normal distribution are presented as mean ± SD, while those without a normal distribution are presented as median (interquartile range). Categorical variables are expressed as frequency (percentage). Comparisons among the three groups based on T2DM statuses (healthy, preDM, and DM) were performed using one-way ANOVA for normally distributed variables (after verifying homogeneity of variance) and the Kruskal-Wallis test for non-normally distributed variables. Pearson’s chi-square test was used to compare categorical variables.

Hardy-Weinberg equilibrium (HWE) was assessed for each VDR polymorphism in the control group using the chi-square test to ensure genotype distributions were consistent with expected frequencies. Deviations from HWE (p < 0.05) were noted and considered in the interpretation of association results.

Logistic regression was conducted to evaluate the association between *VDR* gene polymorphisms (analyzed either as genotypes or carrier states) and T2DM statuses (healthy vs. combined preDM and DM) as the dependent variable, in a crude model and after adjusting for potential confounders based on relevance and literature review. Adjustments were made sequentially as follows: Model 1: crude; Model 2: adjusted for age and gender; Model 3: adjusted for age, gender, and BMI; and Model 4: adjusted for age, gender, BMI, and vitamin D levels. All statistical analyses were performed using Stata, with a p-value < 0.05 considered statistically significant.

## Results

### Basic characteristics of study subjects

Clinical and demographic data of the 976 recruited participants (145 DM, 415 preDM, 416 controls) are presented in Table 1. Our results did not show a significant difference in the gender distribution between the groups (p = 0.06). Age and BMI differed significantly across groups (P < 0.001), with participants with prediabetes or diabetes being older and having higher BMI than healthy controls. Vitamin D levels did not significantly differ among the three groups (p = 0.06).

**Table 1:** Demographic and clinical characteristics of the study population.

Variables		Healthy N = 416	PreDM N = 415	DM N = 145	Total N = 976	P value
**Age**, years Mean (SD)		35.97 (13.39)	45.03 (14.20)	50.04 (12.56)	41.91 (14.64)	<0.001
**BMI**, kg/m^2^ Mean (SD)		25.74 (4.42)	28.03 (4.55)	29.22 (4.26)	27.23 (4.65)	<0.001
**Vitamin D**, ng/ml Median (IQR)		21.2 (14, 33.2)	24 (15.1, 40.7)	25.4 (13.7, 40.3)	23 (14.4, 37.35)	0.06
**Sex**, Number (%)	Men	147 (35.3)	179 (43.1)	54 (37.2)	380 (38.9)	0.06
	Women	269 (64.7)	236 (56.9)	91 (62.8)	596 (61.1)	

preDM: prediabetes mellitus; DM: diabetes mellitus.

### *VDR* gene polymorphism and T2DM

The genotype distributions of the VDR polymorphisms ApaI and FokI in the healthy control group were consistent with HWE (p > 0.05). However, TaqI and EcoRV polymorphisms showed slight deviations from HWE (p = 0.023 and p = 0.041, respectively). The BsmI polymorphism exhibited a strong deviation from HWE (p < 0.00001) as presented in S1 Table. The distributions of the ApaI, TaqI, EcoRV, FokI, and BsmI *VDR* gene polymorphisms (genotypes and carrier states) regarding the T2DM status are presented in Table 2.

**Table 2:** Genotype and carrier state frequencies of VDR polymorphisms by T2DM statuses.

			1. Healthy	2. PreDM	3. DM	Total	P value			
							Overall	1 vs 2	1 vs 3	2 vs 3
**ApaI**	N (%)		401 (100)	409 (100)	143 (100)	953				
	Genotypes	AA	178 (44.39)	155 (37.90)	51 (35.66)	384	0.08	0.07	**0.04**	0.72
		CC	50 (12.47)	71 (17.36)	29 (20.28)	150				
		AC	173 (43.14)	183 (44.74)	63 (44.06)	419				
	A dominant	AA + AC	351 (87.53)	338 (82.64)	114 (79.72)	803	**0.04**	0.05	**0.02**	0.43
		CC	50 (12.47)	71 (17.36)	29 (20.28)	150				
	C dominant	CC + AC	223 (55.61)	254 (62.10)	92 (64.34)	569	0.08	0.06	0.07	0.63
		AA	178 (44.39)	155 (37.90)	51 (35.66)	384				
**TaqI**	N (%)		412 (100)	411 (100)	143 (100)	966				
	Genotypes	CC	158 (38.35)	172 (41.85)	55 (38.46)	385	0.80	0.54	0.83	0.75
		TT	78 (18.93)	69 (16.79)	24 (16.78)	171				
		CT	176 (42.72)	170 (41.36)	64 (44.76)	410				
	T dominant	TT + CT	254 (61.65)	239 (58.15)	88 (61.54)	581	0.55	0.55	0.31	0.98
		CC	158 (38.35)	172 (41.85)	55 (38.46)	385				
	C dominant	CC + CT	334 (81.07)	342 (83.21)	119 (83.22)	795	0.69	0.42	0.57	1.00
		TT	78 (18.93)	69 (16.79)	24 (16.78)	171				
**EcoRV**	N (%)		411 (100)	413 (100)	142 (100)	966				
	Genotypes	CC	57 (13.87)	40 (9.69)	18 (12.68)	115	0.24	0.07	0.90	0.48
		TT	188 (45.74)	179 (43.34)	64 (45.07)	431				
		CT	166 (40.39)	194 (46.97)	60 (42.25)	420				
	T dominant	TT + CT	354 (86.13)	373 (90.31)	124 (87.32)	851	0.17	0.06	0.72	0.32
		CC	57 (13.87)	40 (9.69)	18 (12.68)	115				
	C dominant	CC + CT	223 (54.26)	234 (56.66)	78 (54.93)	535	0.78	0.49	0.89	0.72
		TT	188 (45.74)	179 (43.34)	64 (45.07)	431				
**FokI**	N (%)		411 (42.63)	410 (100)	143 (100)	964				
	Genotypes	TT	20 (4.87)	30 (7.32)	9 (6.29)	59	0.56	0.25	0.61	0.90
		CC	239 (58.15)	222 (54.15)	77 (53.85)	538				
		CT	391 (95.13)	380 (92.68)	134 (93.71)	367				
	C dominant	CC + CT	391 (95.13)	380 (92.68)	134 (93.71)	905	0.34	0.14	0.51	0.68
		TT	20 (4.87)	30 (7.32)	9 (6.29)	59				
	T dominant	TT + CT	172 (41.85)	188 (45.85)	66 (46.15)	426	0.45	0.25	0.37	0.95
		CC	239 (58.15)	222 (54.15)	77 (53.85)	538				
**BsmI**	N (%)		411 (100)	400 (100)	144 (100)	955				
	Genotypes	GG	112 (27.25)	127 (31.75)	44 (30.56)	283	0.59	0.29	0.58	0.94
		AA	28 (6.81)	21 (5.25)	7 (4.86)	56				
		GA	271 (65.94)	252 (63.00)	93 (64.58)	616				
	G dominant	GG + GA	383 (93.19)	379 (94.75)	137 (95.14)	899	0.55	0.35	0.41	0.86
		AA	28 (6.81)	21 (5.25)	7 (4.86)	56				
	A dominant	AA + GA	299 (72.75)	273 (68.25)	100 (69.44)	507	0.36	0.16	0.45	0.79
		GG	112 (27.25)	127 (31.75)	44 (30.56)	196				

preDM: prediabetes mellitus; T2DM: type 2 diabetes mellitus.

**Fig 1:**
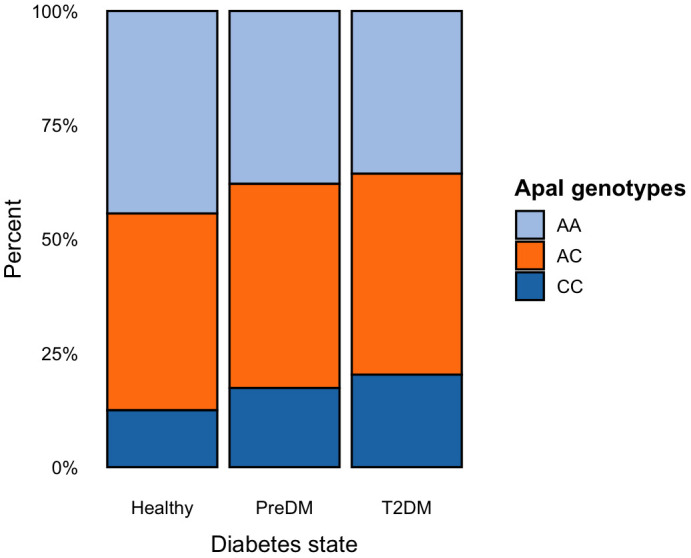
ApaI polymorphism genotype frequencies by diabetes status.

A significant association was observed between the A dominant state of ApaI polymorphism and T2DM statuses (p = 0.044) (Table 2). According to the pair-wise comparisons, the main difference was between healthy and DM participants in both genotype (p = 0.042) and A dominant states (p = 0.023). The *CC* polymorphism was more common in the DM group compared to preDM and healthy individuals, while the *AA* was observed the least in the healthy group (Fig 1). However, there were no significant differences between preDM and DM participants. Regarding other *VDR* gene polymorphisms, no significant association was found between the genotype and dominant states with T2DM statuses.

The association between vitamin D receptor polymorphisms and T2DM statuses was further analyzed across different categories of vitamin D levels. Notably, the association observed for the ApaI polymorphism, under both genotype and dominant genetic models, was present exclusively among individuals with lower vitamin D levels, specifically those with concentrations below either 30 ng/ml (as shown in Table 3) or 50 ng/ml (results not shown). In contrast, no significant associations were identified for other polymorphisms within any vitamin D category.

**Table 3:** The distribution of genotypes and dominant states of ApaI VDR gene polymorphism according to T2DM statuses stratified by vitamin D status.

			1. Healthy	2. PreDM	3. DM	Total	P value
**Vitamin D < 30 ng/ml**							
**ApaI**	N (%)		279 (100)	256 (100)	91 (100)	626	
	Genotypes	AA	123 (44.1)	95 (37.1)	30 (33.0)	248	**0.02**
		CC	36 (12.9)	41 (16.0)	24 (26.4)	101	
		AC	120 (43.0)	120 (46.9)	37 (40.7)	277	
	A dominant	AA + AC	243 (87.1)	215 (84.0)	67 (73.6)	525	**0.01**
		CC	36 (12.9)	41 (16.0)	24 (26.4)	101	
	C dominant	CC + AC	156 (55.9)	161 (63.0)	61 (67.0)	378	0.09
		AA	123 (44.1)	95 (37.1)	30 (33.0)	248	
**Vitamin D ≥ 30 ng/ml**							
**ApaI**	N (%)		122 (100)	153 (100)	52 (100)	327	0.24
	Genotypes	AA	55 (45.1)	60 (39.2)	21 (40.4)	136	
		CC	14 (11.5)	30 (19.6)	5 (9.6)	40	
		AC	53 (43.4)	63 (41.2)	26 (50.0)	142	
	A dominant	AA + AC	108 (88.5)	123 (80.4)	47 (90.4)	278	0.08
		CC	14 (11.5)	30 (19.6)	5 (9.6)	40	
	C dominant	CC + AC	67 (54.9)	93 (60.8)	31 (59.6)	191	0.61
		AA	55 (45.1)	60 (39.2)	21 (40.4)	136	

preDM: prediabetes mellitus; T2DM: type 2 diabetes mellitus.

### Association between ApaI polymorphism and T2DM statuses

Since there were no significant differences in the distribution of ApaI polymorphism between preDM and DM individuals, for logistic regression analysis, we combined the two groups. The logistic regression analysis showed that the ApaI polymorphism is significantly associated with combined preDM and DM (Table 4). The presence of the C alleles is associated with either preDM or DM, with each allele increasing the risk of either preDM or DM by about 29% [OR: 1.29 (1.08, 1.56)]. To be more specific, individuals carrying two *C* alleles (*CC*) are 73% more likely to have either preDM or T2DM compared to those with no *C* alleles (*AA*). The association remained significant after adjusting for other predictors [OR: 1.66 (1.07, 2.56)]. Logistic regression analysis demonstrated that individuals who are carriers of the A allele (*AA* + *AC*) are 36% less likely to develop preDM and DM compared to non-A carriers (*CC*) [OR: 0.64 (0.45, 0.93)]. This association remained significant in the full model as well [OR: 0.65 (0.43, 0.97)]. On the other hand, carriers of the *C* allele (*AC* + *CC*) were 34% more likely to develop preDM or T2DM [OR: 1.34 (1.03, 1.74)] compared to non-*C* carriers (*AA*). However, this association was not significant after adjusting for possible confounders [OR: 1.27 (0.95, 1.69)]. Our findings indicate that the *A* allele is associated with the healthy state.

**Table 4:** The association between ApaI polymorphism (genotypes and dominant states) and T2DM statuses (healthy vs. combined preDM and DM).

	Crude model		Model 2		Model 3		Model 4	
	OR 95%(CI)	P value	OR 95%(CI)	P value	OR 95%(CI)	P value	OR 95%(CI)	P value
**Genotypes**	1.29 (1.08, 1.56)	0.006	1.27 (1.04, 1.55)	0.017	1.26 (1.02, 1.54)	0.028	1.26 (1.02, 1.55)	0.029
**AA**	Ref (1)							
**AC**	1.23 (0.93, 1.62)	0.149	1.19 (0.88, 1.61)	0.254	1.16 (0.85, 1.58)	0.364	1.15 (0.85, 1.58)	0.37
**CC**	1.73 (1.16, 2.56)	0.007	1.69 (1.11, 2.57)	0.015	1.66 (1.08, 2.57)	0.022	1.66 (1.07, 2.56)	0.023
**A dominant**								
**CC**	Ref (1)							
**AA + AC**	0.64 (0.45, 0.93)	0.019	0.65 (0.44, 0.96)	0.03	0.65 (0.43, 0.97)	0.035	0.65 (0.43, 0.97)	0.038
**C dominant**								
**AA**	Ref (1)							
**AC + CC**	1.34 (1.03, 1.74)	0.028	1.30 (0.98, 1.72)	0.066	1.27 (0.95, 1.70)	0.109	1.27 (0.95, 1.69)	0.11

Logistic regression analysis with binary T2DM statuses (healthy vs. combined preDM and DM) as the dependent variable and ApaI genotypes and dominant states as the independent variable. model 1: crude/ model 2: adjusted for age, gender/ model 3: adjusted for age, gender, BMI/ model 4: adjusted for age, gender, BMI, and vitamin D.

Among the five VDR polymorphisms studied, only the ApaI variant demonstrated a significant association with preDM and DM. This association remained significant after adjustment for potential confounders in multivariable logistic regression models. Notably, the ApaI polymorphism was in HWE in the healthy control group, supporting the validity of this finding. In contrast, polymorphisms deviating from HWE (TaqI, EcoRV, and BsmI) did not show significant associations with disease status.

## Discussion

In this study, we used data from the IMOS-3 study to evaluate the association of *VDR* gene polymorphisms (ApaI, TaqI, EcoRV, FokI, and BsmI) with T2DM statuses in a sample of the Iranian population. Our findings revealed a significant link between the ApaI polymorphism and T2DM statuses. According to the logistic analysis, individuals possessing the *CC* genotype were 1.73 times more likely to have either preDM or DM (as combined) compared to those with the *AA* genotype. This suggests that the *C* allele of ApaI may play a role in type 2 Diabetes Mellitus susceptibility. No significant associations were found between the TaqI, EcoRV, BsmI, and FokI polymorphisms and T2DM statuses.

Our results regarding ApaI polymorphism are in line with those of a meta-analysis of 47 case-control studies and 42,812 participants conducted in 2021 to evaluate the association between *VDR* gene polymorphisms and susceptibility to type 2 Diabetes Mellitus [26]. They found a significant association between the ApaI polymorphism and type 2 Diabetes Mellitus in the recessive model (aa vs. Aa + AA) in mixed ethnicity (OR: 1.39 (1.03, 1.87), p = 0.03). However, this association was not significant in the pooled results of subgroup analysis by ethnicity. They also discovered a significant association between FokI, TaqI, and BsmI polymorphisms in the heterozygote model in Asians. The ApaI variable site (*C* > A*)* is located in intron 8 of the *VDR* gene, and although non-functional, it is associated with a poly (A) microsatellite repeat in the 3’ untranslated region (UTR), which can impact disease development by influencing the stability of *VDR* mRNA [27]. Reduced mRNA stability, leading to lower *VDR* protein levels, may result in a weaker vitamin D response. In this context, the ApaI polymorphism might act as an intronic enhancer, potentially altering the splicing of *VDR* mRNA or enhancing gene transcription [28]. However, the functional relevance of ApaI in type 2 Diabetes Mellitus remains uncertain and should be explored in future studies.

Another study on Iranian participants showed that *CCC* and *TCC VDR* haplotypes of ApaI, TaqI, and EcoRV polymorphisms are risk factors for diabetic nephropathy in patients with T2DM [23]. A study of Iranian pregnant women found that the *CC* genotype of the ApaI polymorphism increased the risk of gestational diabetes mellitus compared to the AA genotype (P = 0.012) [29]. However, in contrast to our findings, there are several studies finding no significant associations [14,19,30–34]. In a case-control study including 730 Iranian subjects, no association between BsmI, ApaI, TaqI, or FokI polymorphisms and the risk of type 2 Diabetes Mellitus was seen [20]. This lack of association between all four SNPs and type 2 Diabetes Mellitus was also seen in Indian, Emirati, and Brazilian populations [31,17,35].

We did not observe any association between the FokI polymorphism and T2DM. Similar trends were observed in some studies [12,14,34,36–40], though findings vary across populations and many studies did not match our findings and reported an association [19,20,31,41]. A study of Iranian individuals with type 2 Diabetes Mellitus, comparing those with and without diabetic foot ulcers (DFU), revealed an association between the FokI polymorphism and diabetic foot ulcer [22]. FokI is unique among the polymorphisms assessed in our study as it is located within the gene’s coding sequence and results in different *VDR* protein isoforms [39]. The FokI SNP is characterized by a *T > C* substitution at the start codon of exon 2, which results in a shorter protein with 424 amino acids instead of 427, affecting the receptor’s biological activity [42]. Given this context, FokI seems to have potential in predicting the risk of type 2 Diabetes Mellitus and can be a target of future studies involving larger populations and different ethnicities.

Considering potential ethnic variations, findings from most previous studies likewise show no significant association between the TaqI polymorphism and type 2 Diabetes Mellitus [14,19,20,31–33,37]; however, a few reports have indicated a possible association [43,44]. A case-control study in the Kashmiri population found that the T allele in TaqI and the *G* allele in BsmI polymorphisms might make individuals susceptible to type 2 Diabetes Mellitus [43]. The association of the BsmI polymorphism with type 2 Diabetes Mellitus is also debated; some studies support our findings of no association [19,20,31,37,12,39,41], while others report a significant association [34,45]. A 2023 study in an Iranian population found that the *C* allele and *CC* genotype of the TaqI polymorphism were risk factors for developing type 2 Diabetes Mellitus, whereas BsmI did not show a significant association with the disease [21].

Our study, which is among a few studies that have explored the link between the EcoRV (*T > C*) polymorphism and developing type 2 Diabetes Mellitus, did not reveal a significant association [46]. Another study conducted on the IMOS data found that the *TT* genotype of the EcoRV polymorphism increased the risk of low bone density and osteopenia in women with T2DM compared to the *CT* genotype [47]. A similar result was observed in a recent study conducted in an Iranian population in which the T allele showed a significant association with susceptibility to osteoporosis [48]. EcoRV (rs4516035, also called GATA) is located in the 5′ regulatory region of chromosome 12 [46]. According to in vitro studies, the *C* allele of the EcoRV polymorphism has a transcription rate that is two times lower than that of the *T* allele, which may result in a decrease in the expression of the *VDR* [46].

We didn’t find a significant difference in vitamin D levels between type 2 Diabetes Mellitus patients and controls in this study (p = 0.06), while the literature mostly points to reduced vitamin D in individuals with the disease [49–52]. This can be explained by several factors. Firstly, Iran has a high prevalence of vitamin D deficiency across the general population, with systematic reviews reporting deficiency rates around 45–62% in men and women, and even higher rates in pregnant women and certain urban areas [53,54]. A high prevalence of vitamin D deficiency and insufficiency (Vit D < 30 ng/ml) was also seen in our study (65.7%). This widespread deficiency means that both diabetic and non-diabetic groups may have similarly low vitamin D levels, minimizing detectable differences. Secondly, seasonal variation in sampling time can affect measured vitamin D levels, and differences may be obscured without controlling for seasonality. Moreover, differences in ethnicity, assay type, vitamin D cutoff definitions, vitamin D supplementation, population dietary habits, study methodologies, and potential publication bias toward positive findings may also contribute to these inconsistencies.

Our study also does not show an association between *VDR* gene polymorphisms and vitamin D levels, which is in line with some other studies [55,56]. One possible explanation is that the pathogenesis of type 2 Diabetes Mellitus involves complex genetic, endocrine, and environmental factors, making it difficult to isolate the impact of VDR alone [42]. Additionally, the role of inflammatory processes and immune system activation in type 2 Diabetes Mellitus may overshadow the effects of vitamin D in insulin secretion and glucose intolerance [42,57]. Although vitamin D has anti-inflammatory properties, many other SNPs affecting cytokine levels have also been linked to the disease [58,59]. Moreover, the impact of *VDR* polymorphisms on type 2 Diabetes Mellitus is further complicated by gene-gene and gene-environment interactions. Our results regarding the association between ApaI polymorphism and T2DM statuses remained significant even after adjusting for cofounding factors, including vitamin D levels, suggesting that *VDR* gene variations might affect type 2 Diabetes Mellitus susceptibility through pathways beyond vitamin D levels. However, in the subgroup analysis by vitamin D deficiency, the association between ApaI polymorphism and diabetes status lost significance in both the groups with vitamin D levels above 30 and 50 ng/ml. Nevertheless, it should be noted that these groups had a smaller population compared to the population with vitamin D levels below either 30 or 50 ng/ml, and therefore, reduced statistical power.

### Strengths and limitations

IMOS is a national, population-based study, making our results a valuable addition to the literature, particularly concerning the Iranian population. We investigated 5 SNPs, including the less-studied EcoRV, making our study one of the most comprehensive assessments of the *VDR* gene in relation to T2DM. Moreover, we enhanced the robustness of our findings by adjusting for multiple variables, including vitamin D levels. Additionally, we evaluated the association of SNPs with preDM, offering insights that can help implement early preventive strategies. However, some limitations of our study should be considered, such as its cross-sectional design, which prevents us from concluding causality. Also, the polymorphisms selected in our study do not provide complete coverage of the SNPs in the *VDR* gene [38,60,61], so we cannot rule out the possibility that other *VDR* variants may be associated with T2DM. Comprehensive analysis of all known *VDR* polymorphisms and their interactions is crucial, as the interplay between promoter, coding, and 3’ UTR polymorphisms significantly influences *VDR* expression and activity [27,62]. The significant association observed for the ApaI polymorphism with preDM and DM, alongside its conformity to HWE in controls, strengthens the evidence for its potential role in disease susceptibility in this Iranian population.

We observed a slight deviation from HWE for TaqI and EcoRV and a strong deviation for BsmI. These deviations may be attributed to the random sample clustering method, selection bias, or technical genotyping factors. While we partly addressed this through regression analysis, the potential impact of these deviations should be considered when interpreting the results. Consequently, we focused our primary interpretation on the ApaI variant, which demonstrated both genetic stability and a robust association with the outcome after adjusting for confounders.

## Conclusion

Our results revealed a significant association between the ApaI polymorphism and type 2 Diabetes Mellitus susceptibility in a sample of the Iranian population. The presence of the C allele may be associated with increased susceptibility to prediabetes and type 2 Diabetes Mellitus, with the *CC* genotype posing the highest risk. However, no significant associations were found between TaqI, EcoRV, BsmI, or FokI polymorphisms and progression to T2DM. Understanding these genetic factors could contribute to personalized prevention and management strategies for T2DM. More large-scale studies, particularly among diverse ethnic groups, are needed to validate these findings and investigate the underlying mechanisms.

## Supporting information

S1 Table Hardy-Weinberg equilibrium analysis of genotype frequencies in healthy controls for VDR polymorphisms. (DOCX)
