# Supplementary material for: Association of VDR gene polymorphisms with prediabetes and Type 2 diabetes mellitus in a sample of the Iranian population
Source: PLoS One. 2026 Feb 9;21(2):e0339758. doi: 10.1371/journal.pone.0339758 (PMC12885287; doi:10.1371/journal.pone.0339758)
Supplement: S1 Table — (DOCX) [file pone.0339758.s001.docx]

S1 Table. Hardy-Weinberg Equilibrium Analysis of Genotype Frequencies in Healthy Controls for VDR Polymorphisms

| **Polymorphism** | **Genotype (Observed count, %)** | **Expected Count (HWE)** | **Chi-square** | **p-value** | **Deviation from HWE?** |
| --- | --- | --- | --- | --- | --- |
| ApaI | AA: 178 (44.39%) AC: 173 (43.14%) CC: 50 (12.47%) | AA: 174.46 AC: 180.07 CC: 46.46 | 0.618 | 0.4317 | No |
| TaqI | CC: 158 (38.35%) CT: 176 (42.72%) TT: 78 (18.93%) | CC: 146.88 CT: 198.23 TT: 66.88 | 5.183 | 0.0228 | mild |
| EcoRV | CC: 57 (13.87%) CT: 166 (40.39%) TT: 188 (45.74%) | CC: 47.69 CT: 184.62 TT: 178.69 | 4.182 | 0.0409 | mild |
| FokI | CC: 239 (58.15%) CT: 152 (36.98%) TT: 20 (4.87%) | CC: 241.42 CT: 147.15 TT: 22.42 | 0.446 | 0.5043 | No |
| BsmI | AA: 28 (6.81%) GA: 271 (65.94%) GG: 112 (27.25%) | AA: 65.04 GA: 196.92 GG: 149.04 | 58.174 | 0.0000 | significant |
